# Supplementary material for: Baseline Characterization of the Gut Microbiota of Field and Colony Populations of Phlebotomus tobbi and Preliminary Assessment of the Anti-Leishmanial Activity of Cultivable Bacteria
Source: Pathogens. 2026 Jun 23;15(7):658. doi: 10.3390/pathogens15070658 (PMC13416292; doi:10.3390/pathogens15070658)
Supplement: Supplementary file 1 [file pathogens-15-00658-s001.zip › pathogens-4357168-File S1/Heatmap/5.family/heatmap_without_sample_clustering.pdf]

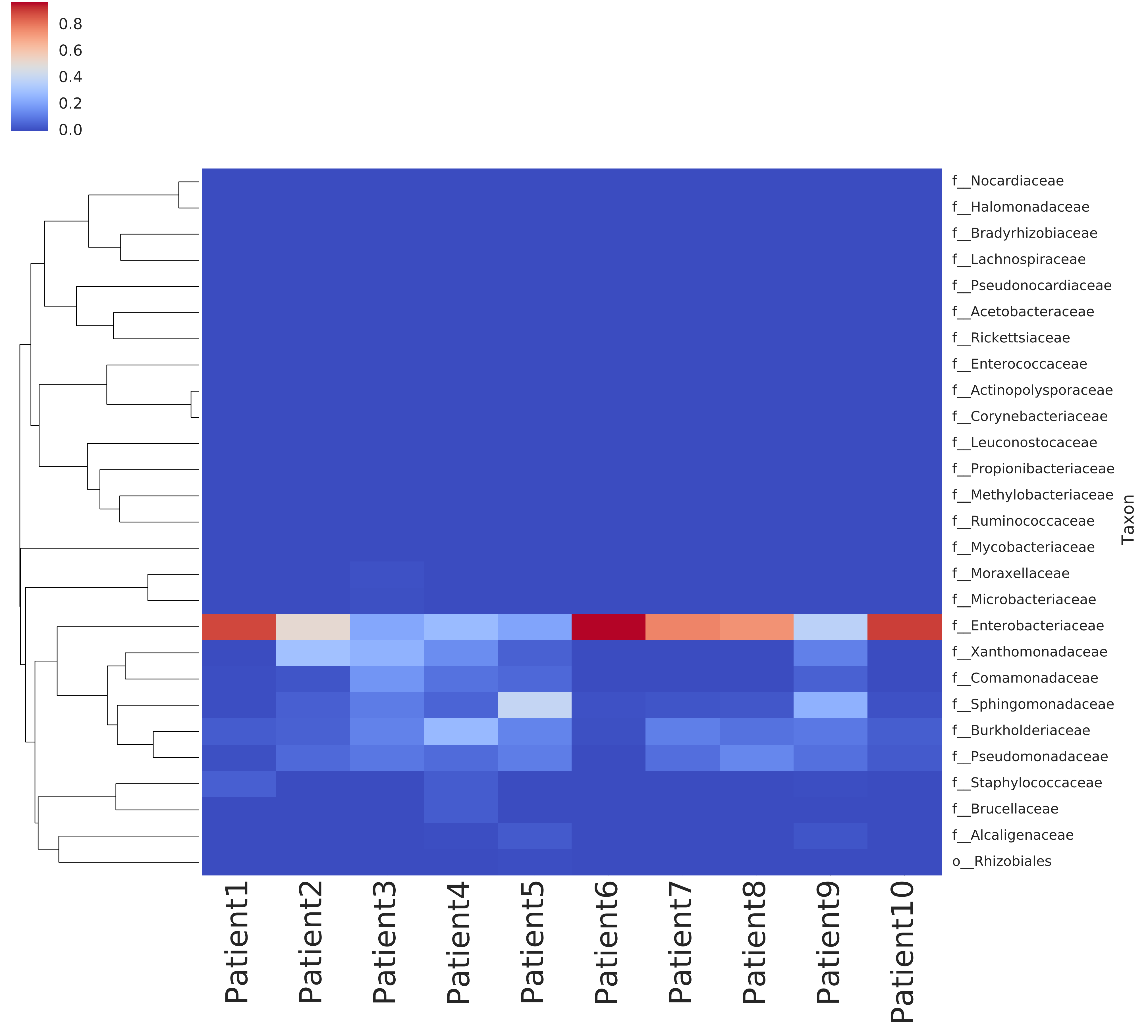
0.8
0.6
0.4
0.2
0.0
f__Nocardiaceae
f__Halomonadaceae
f__Bradyrhizobiaceae
f__Lachnospiraceae
f__Pseudonocardiaceae
f__Acetobacteraceae
f__Rickettsiaceae
f__Enterococcaceae
f__Actinopolysporaceae
f__Corynebacteriaceae
f__Leuconostocaceae
f__Propionibacteriaceae
f__Methylobacteriaceae
f__Ruminococcaceae
f__Mycobacteriaceae
f__Moraxellaceae
f__Microbacteriaceae
f__Enterobacteriaceae
f__Xanthomonadaceae
f__Comamonadaceae
f__Sphingomonadaceae
f__Burkholderiaceae
f__Pseudomonadaceae
f__Staphylococcaceae
f__Brucellaceae
f__Alcaligenaceae
o__Rhizobiales
Taxon
Patient1
Patient2
Patient3
Patient4
Patient5
Patient6
Patient7
Patient8
Patient9
Patient10
